# Texture analysis on the edge-enhanced fluence of VMAT

**DOI:** 10.1186/s13014-015-0382-z

**Published:** 2015-04-01

**Authors:** So-Yeon Park, Jong Min Park, Wonmo Sung, Il Han Kim, Sung-Joon Ye

**Affiliations:** Interdiciplinary Program in Radiation Applied Life Science, Seoul National University College of Medicine, Seoul, Republic of Korea; Department of Radiation Oncology, Seoul National University Hospital, Seoul, Republic of Korea; Biomedical Research Institute, Seoul National University College of Medicine, Seoul, Republic of Korea; Institute of Radiation Medicine, Seoul National University Medical Research Center, Seoul, Republic of Korea; Center for Convergence Research on Robotics, Advanced Institutes of Convergence Technology, Suwon, Republic of Korea; Department of Transdisciplinary Studies, Program in Biomedical Radiation Sciences, Seoul National University Graduate School of Convergence Science and Technology, Seoul, Republic of Korea; Department of Radiation Oncology, Seoul National University College of Medicine, Seoul, Republic of Korea

**Keywords:** Texture analysis, Volumetric modulated arc therapy, Modulation index, Fluence

## Abstract

**Background:**

Textural features of edge-enhanced fluence were analysed to quantify modulation degree of volumetric modulated arc therapy (VMAT) plans.

**Methods:**

Twenty prostate and twenty head and neck VMAT plans were retrospectively selected. Fluences of VMAT plans were generated by integration of monitor units shaped by multi-leaf collimators (MLCs) at each control point. When generating fluences, the values of pixels representing MLC tips were doubled to prevent smearing out of small or irregular fields (edge-enhancement). Six kinds of textural features, including *angular second moment*, *inverse difference moment*, *contrast*, *variance*, *correlation* and *entropy,* were calculated with particular displacement distances (*d*) of 1, 5 and 10. Plan delivery accuracy was evaluated by gamma-index method, mechanical parameter differences between plan and delivery and differences in dose-volumetric parameters between plan and delivery. Spearman’s correlation coefficients (*r*_*s*_) were calculated between the values of textural features and VMAT delivery accuracy.

**Results:**

The *r*_*s*_ values of *contrast* (*d* = 1) with edge-enhancement to global gamma passing rates with 2%/2 mm, 1%/2 mm and 2%/1 mm were 0.546 (*p* < 0.001), 0.744 (*p* < 0.001) and 0.487 (*p* = 0.001), respectively. Those with local 2%/2 mm, 1%/2 mm and 2%/1 mm were 0.588, 0.640 and 0.644, respectively (all with *p* < 0.001). The *r*_*s*_ values of *contrast* (*d* = 1) to MLC and gantry angle errors were -0.853 and 0.655, respectively (all with *p* < 0.001). The *contrast* (*d* = 1) showed statistically significant *r*_*s*_ values in 11 dose-volumetric parameter differences from a total of 35 cases, and generally showed better correlations to plan delivery accuracy than did previously suggested textural features with non-edge-enhanced fluences, as well as conventional modulation indices.

**Conclusions:**

*Contrast* (*d* = 1) with edge-enhanced fluences could be used as modulation index for VMAT.

## Background

Volumetric modulated arc therapy (VMAT) enables rapid delivery of intensity-modulated photon beams by simultaneous modulations of mechanical parameters, *i.e.* multi-leaf collimator (MLC) positions, gantry rotation speed and dose-rate [1,2]. Since VMAT can deliver comparable or better dose distributions to a patient faster than intensity-modulated radiation therapy (IMRT), it has been widely adopted in the clinic [2-4]. However, just as with IMRT, excessive modulation of photon beam intensity of VMAT results in discrepancies in the dose distributions between treatment plans and actual delivery due to increased uncertainty in the mechanical operation of the linac [5,6]. Excessive modulation may also lead to increased small or irregular field usage, which can potentially cause inaccurate calculation of dose distributions in commercial treatment planning systems (TPS) [5,6]. Therefore, various verification methods have been suggested for IMRT and VMAT since they were introduced in the field of radiation therapy [7-13].

Pre-treatment quality assurance (QA) using the gamma-index method with a planar dose distribution measured using a detector array is a popular verification method for both IMRT and VMAT, and widely used clinically [7,13-15]. However, several recent studies have demonstrated the weak clinical relevance of gamma passing rates [8,16-18]. Nelms *et al.* demonstrated weak correlations between gamma passing rates and anatomy-based dose-volumetric parameters by introducing intentional errors in IMRT plans [16]. Another option for plan verification that has been suggested is the analysis of log files registered by the linac control system during IMRT or VMAT [8,9,12,18]. This method is limited in that it is not an independent verification system of delivery. Calculation based methods, such as modulation indices, for verifying the accuracy of IMRT or VMAT plan delivery have also been suggested [5,6,19-23]. For VMAT, Masi *et al.* suggested the modulation complexity score for VMAT (MCS_v_) and the leaf travel modulation complexity score (LTMCS) by modifying modulation complexity score (MCS) which was originally suggested by McNiven *et al*. for IMRT [20,22]. Li and Xing suggested another modulation index for VMAT (MI_SPORT_) by quantifying MLC positional variations weighted by segmental monitor units (MU) at each control point (CP) [21]. Those studies tried to quantify modulation degree of VMAT with variations of MLC positions. Park *et al.* suggested modulation index (MI_t_) by quantifying MLC speeds, MLC accelerations, gantry rotation accelerations and dose-rate variations simultaneously [6]. Considerable correlations between the values of MI_t_ and the gamma passing rates as well as the results of linac log file analysis were shown with statistical significances in that study. We analysed textural features calculated from fluences of VMAT plans to quantify the modulation degree of VMAT in a previous study [23]. In that study, textural features were calculated from a single fluence per VMAT plan. The fluence was generated by integration of all MUs shaped by MLC apertures (MU maps) at each CP. Although we demonstrated considerable correlations of textural features to the discrepancy between plan and delivery, some small or irregular fields at different CPs could be potentially smeared out when fluences were generated by the whole integration of various shaped MU maps. For example, if several small fields with the same MU, which could potentially cause discrepancy between plan and delivery, are contained in a VMAT plan at different CPs, and if those small fields make a single large field when they are integrated, those small fields cannot be identified with a fluence generated by the whole integration of every MU map. If we can distinguish every small field in a fluence, textural features calculated with that fluence might have better power to quantify the modulation degree of VMAT.

In this study, we tried to distinguish all the small fields in the fluence of a VMAT plan in order to calculate textural features considering every aperture at each CP. To this end, we enhanced the values of edges shaped by MLCs at each CP in a fluence. We tested the performance of the textural features calculated with edge-enhanced fluences using correlation analysis, and compared the results to the indices suggested in our previous study, as well as to the conventional set of modulation indices suggested for VMAT [6,21-23].

## Methods

### Sampling of VMAT plans

Twenty VMAT plans for head and neck (H&N) cancer and twenty VMAT plans for prostate cancer which were selected in our previous study were used again for this study to compare textural features calculated with edge-enhanced fluences to those calculated with non-enhanced fluences [23]. All VMAT plans were generated with 6 MV photon beams of Trilogy™ with Millennium™ 120 MLC (Varian Medical Systems, Palo Alto, CA) and used two full arcs. All the VMAT plans were optimized with the progressive resolution optimizer 3 (PRO3, ver.10, Varian Medical Systems, Palo Alto, CA) and dose distributions were calculated with the anisotropic analytic algorithm (AAA, ver.10, Varian Medical Systems, Palo Alto, CA) in the Eclipse™ system (Varian Medical Systems, Palo Alto, CA). The dose calculation grid of patient CT images was 2.5 mm. All VMAT plans were clinically acceptable, showing global gamma passing rates with gamma criterion of 2%/2 mm of higher than 90% as recommended by Heilemann *et al* [13]. In our institution, prostate cancer is treated with sequential delivery of a primary plan delivering 50.4 Gy to both the prostate and seminal vesicles in 28 fractions, and a boost plan delivering 30.6 Gy to the prostate in 17 fractions. Primary plans were analysed in this study. For H&N VMAT plans, prescription doses of 67.5 Gy, 54 Gy and 48 Gy were delivered to a total of 3 target volumes in 30 fractions with simultaneous integrated boost (SIB) technique.

### Fluence generation with edge-enhancement

Each VMAT plan was exported in DICOM-RT format from the Eclipse™ system. Using an in-house program written in MATLAB (ver.8.1, Mathworks, Inc., Natick, MA), fluences for each plan were generated by integration of every MU shaped by the MLCs at each CP. Although the width of Millennium™ 120 MLC is 5 mm in the central region, and 10 mm for periphery region, the resolution of the fluences was set to be 1 mm for detailed analysis in the direction of MLC movement. When integrating MU maps to make a single fluence for each VMAT plan, the values (MUs) of pixels (size of 1 mm × 1 mm) representing MLC tips were doubled (edge-enhancement of fluence). In other words, the field apertures defined by MLC tips were highlighted by doubling the values of pixels representing MLC tips at each CP. The goal of this was to distinguish individual small fields at different CPs contained in a single VMAT plan. By doing this, we could reduce the probability of smearing out of some small fields at different CPs when generating a fluence which was a superposition of every MU map at each CP. Unlike IMRT, MLCs of VMAT moves in and out continuously during beam delivery [24], therefore, if we enhance the edges of MU maps parallel to the direction of MLC movement, excessively high values would be assigned at that region in a fluence. Since this could be a disturbance factor of texture analysis, and small (or irregular) fields could be identified without edge-enhancement of this region, the edges parallel to the MLC moving direction were not enhanced, but the edges perpendicular to the direction of MLC movement (*i.e.* MLC tip) were enhanced. Consequently, the edge-enhanced fluences showed a lot of short discrete lines perpendicular to the direction of MLC movement, in contrast to the relatively smoother fluences without edge-enhancement. The length and width of those lines were 5 mm (or 10 mm) by 1 mm due to the width of MLCs and the resolution of fluence in this study, respectively.

### Calculation of textural features

The methods used to calculate textural features were the same as those in our previous study [23]. The difference in textural features between this study and our previous study was that textural features in this study were calculated with edge-enhanced fluences. First, gray level co-occurrence matrices (GLCMs) were calculated from each edge-enhanced fluence of VMAT plans in order to calculate textural features. The GLCM is a matrix or distribution indicating the co-occurring values at a given offset (*i.e.* particular displacement distance, *d*) [23,25,26]. When finding co-occurring values, the angles of searching directions were 0°, 45°, 90° and 135° for each value of *d.* The values of *d* in this study were 1, 5 and 10. Since the resolution of the fluences in this study was 1 mm, the fluence was investigated at the distance of 1 mm, 5 mm and 10 mm in the horizontal and vertical directions ($$ \sqrt{2}, $$$$ 5\sqrt{2} $$ and $$ 10\sqrt{2} $$ for diagonal directions). Just as in our previous study, textural features such as *angular second moment* (*ASM*), *inverse difference moment* (*IDM*), *contrast*, *variance*, *correlation* and *entropy* were calculated with from the GLCM [23]. Since 6 kinds of textural features were calculated with 3 values of *d* (1, 5 and 10), a total of 18 textural features were calculated for each VMAT plan.

### Quantification of plan delivery accuracy of VMAT

The data indicating VMAT plan delivery accuracy were the same as those in our previous study [23]. Three kinds of methods for each VMAT plan were adopted to verify VMAT plan delivery accuracy, which were the gamma-index method with a planar dose distribution, mechanical parameter differences between original treatment plan and linac log files registered during delivery, and differences in dose-volumetric parameters of each organ at risk (OAR) as well as target volumes between original treatment plan and the plan reconstructed with linac log files registered during delivery.

For the gamma-index method, the calculated planar dose distribution in the Eclipse™ system was compared to the dose distributions measured using a MapCHECK2™ detector array (Sun Nuclear Corporation, Melbourne, FL) inserted in the MapPHAN™ (Sun Nuclear Corporation, Melbourne, FL). For accurate measurements, the output of the linac was calibrated based on American Association of Physicists in Medicine (AAPM) Task Group (TG) 51 protocol and the readings of detectors in the MapCHECK2™ detector array were calibrated following the guidelines provided by the manufacturer, before measurements of planar dose distributions [27]. For exact setup of the device, a cone beam computed tomography (CBCT) image of the device was taken and setup was corrected by matching the CT images and CBCT images of the device before measurements. Both global and local gamma evaluations were performed with gamma criteria of 2%/2 mm, 1%/2 mm and 2%/1 mm. Following recommendations of previous studies on the gamma-index method for VMAT, gamma criteria of 3%/3 mm and 1%/1 mm were not used in this study [13,28]. Since we used gamma criterion of 2%/1 mm, the calculation grid of planar dose distribution in the Eclipse™ system was set to be 1 mm. The points of doses less than 10% of the maximum dose were not evaluated as often cited in the literature [13,15,29,30].

During measurements of planar dose distributions, both dynamic log files and DynaLog files were acquired for each VMAT plan. The information of gantry angles and delivered MUs at each CP during delivery was acquired from dynamic log files while the information of MLC positions was acquired from DynaLog files. With an in-house program written in MATLAB, dynamic log files and DynaLog files were combined into DICOM-RT format. After that, the differences in MLC positions, gantry angles and delivered MUs between original treatment plans and those recorded during delivery were calculated at each CP and averaged for each VMAT plan.

The DICOM-RT format files were imported into the Eclipse™ system and dose distributions were calculated with patient CT images under the same conditions as treatment planning for patient treatment. The differences in the values of dose-volumetric parameters between original treatment plans and plans recalculated using log files were calculated. As dose-volumetric parameters for target volumes, the dose received by 95% of target volume (D_95%_), D_5%_, the minimum, maximum and mean dose were calculated. For OARs of prostate VMAT plans, D_20%_ and mean dose to rectal wall, D_20%_ and mean dose to bladder and D_50%_ and mean dose to femoral heads were calculated. For OARs of H&N VMAT plans, mean dose to each parotid gland and the maximum dose to the spinal cord, brain stem, each lens, optic chiasm and each optic nerve were calculated [23].

### Correlation analysis

To investigate the correlation of the values of textural features to the VMAT plan delivery accuracy, correlation analysis between the textural features and results of 3 kinds of VMAT verification methods mentioned above was performed individually. Spearman’s rank correlation coefficients (*r*_*s*_) and corresponding *p* values were calculated. The *p* values were calculated under the two-tailed unpaired parameter condition.

## Results

### Values of textural features

**Figure 1:**
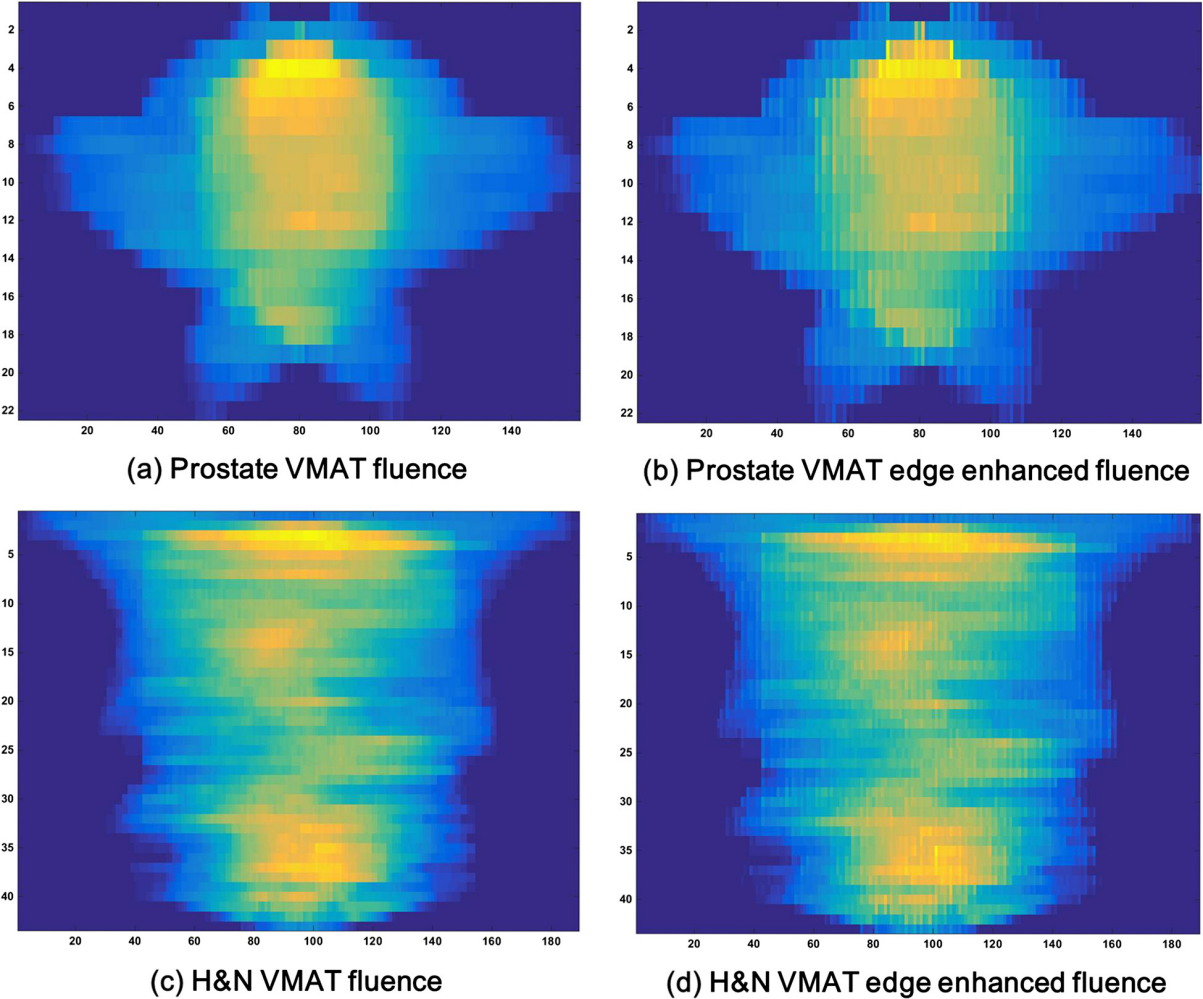
**Edge-enhanced and non-enhanced fluence of VMAT.** The fluences with non-edge-enhancement of prostate **(a)** and head and neck (H&N) volumetric modulated arc therapy (VMAT) plans **(c)** are shown. Those fluences were generated by whole integration of every monitor units (MUs) shaped by multi-leaf collimator (MLC) apertures at each control point (CP). The fluences with edge-enhancement of prostate **(b)** and H&N VMAT plans **(d)** are also shown. For edge-enhancement of fluences, when integrating MUs, the values of pixels (size of 1 mm × 1 mm) representing MLC tips were doubled.

**Figure 2:**
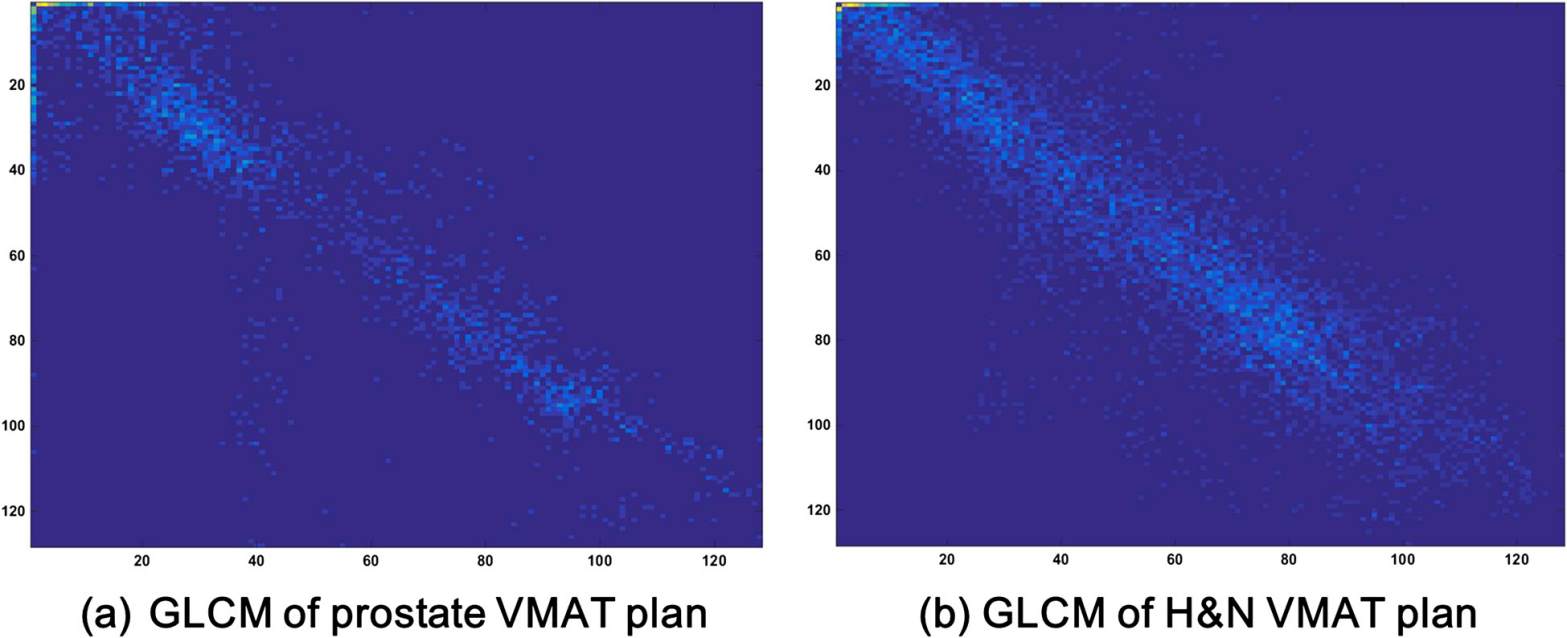
**Gray level co-occurrence matrix of VMAT fluence.** The gray level co-occurrence (GLCM) matrices generated with edge-enhancement of prostate **(a)** and H&N VMAT plans **(b)** are shown. The particular displacement distance (*d*) was 1 and the searching angles were 0°, 45°, 90° and 135° when generating GLCM from a fluence.

The fluences with and without edge-enhancement of prostate and H&N VMAT plans are shown in Figure 1. The GLCMs generated with edge-enhancement of those prostate and H&N VMAT plans are shown in Figure 2. The textural features of prostate and H&N VMAT plans calculated from the GLCMs, and *p* values showing the statistical significances of their differences are shown in Table 1. All textural features of prostate VMAT plans were different from those of H&N VMAT plans with statistical significances (all with *p* < 0.003). The values of *ASM*, *contrast* and *variance* of prostate VMAT plans were higher than those of H&N VMAT plans, while the values of *IDM*, *correlation* and *entropy* of H&N VMAT plans were higher than those of prostate VMAT plans. This tendency of the values of textural features calculated with edge-enhanced fluences was the same as that of the textural features with non-edge-enhanced fluences in our previous study, although the values were different from each other [23].

**Table 1:** **The values of textural features calculated from edge-enhanced fluences**

	***d*** **= 1**			***d*** **= 5**			***d*** **= 10**		
	**Prostate**	**H&N**	***p***	**Prostate**	**H&N**	***p***	**Prostate**	**H&N**	***p***
ASM (×10^−3^)	1.433 ± 0.170	0.842 ± 0.264	<0.001	2.183 ± 0.497	0.837 ± 0.270	<0.001	2.972 ± 0.650	0.989 ± 0.439	<0.001
IDM	0.236 ± 0.021	0.271 ± 0.027	<0.001	0.115 ± 0.013	0.167 ± 0.020	<0.001	0.082 ± 0.011	0.126 ± 0.015	<0.001
Contrast	264.73 ± 70.21	113.52 ± 51.79	<0.001	960.75 ± 236.74	341.90 ± 152.29	<0.001	1737.04 ± 484.32	537.56 ± 239.04	<0.001
Variance	45.48 ± 4.60	37.48 ± 7.74	0.001	46.41 ± 4.49	38.59 ± 8.08	0.001	46.32 ± 4.64	39.06 ± 8.10	0.002
Correlation	0.874 ± 0.021	0.921 ± 0.017	<0.001	0.583 ± 0.050	0.791 ± 0.035	<0.001	0.308 ± 0.132	0.686 ± 0.059	<0.001
Entropy	2.971 ± 0.056	3.243 ± 0.118	<0.001	2.875 ± 0.071	3.308 ± 0.124	<0.001	2.730 ± 0.092	3.287 ± 0.151	<0.001

*Abbreviations:* d = particular displacement distance, prostate = volumetric modulated arc therapy plans for prostate cancer, H&N = volumetric modulated arc therapy plans for head and neck cancer, ASM = angular second moment, IDM = inverse difference moment.

### Correlations between textural features and gamma passing rates

The values of both global and local gamma passing rates of the 40 VMAT plans are shown in our previous study [23]. The values of *r*_*s*_ and corresponding *p* values of each textural feature to global gamma passing rates are shown in Table 2. With the exceptions of *r*_*s*_ values between global 2%/2 mm and *entropy* (*d* = 1), global 2%/1 mm and *ASM* (d = 1, 5 and 10), and *correlation* (*d* = 1, 5 and 10) and *entropy* (*d* = 1, 5 and 10), every value of *r*_*s*_ was statistically significant, showing *p* values less than 0.05. The highest correlation was observed in *contrast* (*d* = 1) and global 1%/2 mm (*r*_*s*_ = 0.744 with *p* < 0.001). The values of *r*_*s*_ of *contrast* (*d* = 1) calculated with edge-enhanced fluences to global gamma passing rates were generally higher than those of *contrast* (*d* = 1) and *variance* (*d* = 1) with non-edge-enhanced fluences, which showed the best performance in our previous study [23]. In addition, the values of *r*_*s*_ of *contrast* (*d* = 1) with edge-enhanced fluences to global gamma passing rates were always higher than those of MCS_v_, LTMCS and MI_SPORT_. *Contrast* (*d* = 1) with edge-enhancement showed higher values of *r*_*s*_ than MI_t_ to passing rates with 2%/2 mm (0.546 with *p* < 0.001 for *contrast* vs. -0.536 with *p* < 0.001 for MI_t_) and 2%/1 mm (0.487 with *p* < 0.001 for *contrast* vs. -0.361 with *p* = 0.022 for MI_t_). However, it showed lower value of *r*_*s*_ to passing rates with 1%/2 mm than MI_t_ (0.744 with *p* < 0.001 for *contrast* vs. -0.764 with *p* < 0.001 for MI_t_).

**Table 2:** **The values of r**_**s**_ **between textural features and global gamma passing rates**

		**2%/2 mm**		**1%/2 mm**		**2%/1 mm**	
	***d***	***r***_***s***_	***p***	***r***_***s***_	***p***	***r***_***s***_	***p***
ASM	1	0.373	0.018	0.623	<0.001	0.174	0.282
	5	0.400	0.011	0.569	<0.001	0.133	0.415
	10	0.381	0.015	0.571	<0.001	0.187	0.249
IDM	1	−0.610	<0.001	−0.690	<0.001	−0.503	0.001
	5	−0.582	<0.001	−0.713	<0.001	−0.417	0.007
	10	−0.540	<0.001	−0.668	<0.001	−0.362	0.022
Contrast	1	0.546	<0.001	0.744	<0.001	0.487	0.001
	5	0.570	<0.001	0.725	<0.001	0.450	0.004
	10	0.554	<0.001	0.740	<0.001	0.402	0.010
Variance	1	0.486	0.001	0.623	<0.001	0.485	0.002
	5	0.507	0.001	0.630	<0.001	0.490	0.001
	10	0.486	0.001	0.587	<0.001	0.530	<0.001
Correlation	1	−0.390	0.013	−0.648	<0.001	−0.279	0.082
	5	−0.486	0.001	−0.678	<0.001	−0.247	0.125
	10	−0.374	0.017	−0.633	<0.001	−0.093	0.569
Entropy	1	−0.303	0.057	−0.581	<0.001	−0.210	0.194
	5	−0.363	0.021	−0.594	<0.001	−0.201	0.213
	10	−0.312	0.050	−0.597	<0.001	−0.200	0.216

*Abbreviations:* d = particular displacement distance, r_s_ = Spearman’s rank correlation coefficient, ASM = angular second moment, IDM = inverse difference moment.

The values of *r*_*s*_ and corresponding *p* values of each textural feature to local gamma passing rates are shown in Table 3. All values of *r*_*s*_ to local gamma passing rates were statistically significant, showing *p* values less than 0.05. The highest value of *r*_*s*_ was observed between *contrast* (*d* = 1) and gamma passing rates with 2%/1 mm (*r*_*s*_ = 0.644 with *p* < 0.001). *Contrast* (*d* = 1) always showed higher values of *r*_*s*_ with statistical significances than the other textural features to local gamma passing rates with every gamma criterion tested in this study. The values of *r*_*s*_ of *contrast* (*d* = 1) with edge-enhanced fluences were always higher than those of *contrast* (*d* = 1) and *variance* (*d* = 1) with non-edge-enhanced fluences [23]. In addition, *contrast* (*d* = 1) with edge-enhanced fluences always showed higher correlations than did MCS_v_, LTMCS and MI_SPORT_. However, compared to MI_t_, *contrast* (*d* = 1) with edge-enhanced fluences showed higher correlations to local gamma passing rates with 2%/1 mm, but lower values of *r*_*s*_ to gamma passing rates with 2%/2 mm and 1%/2 mm than did MI_t_.

**Table 3:** **The values of r**_**s**_ **between textural features and local gamma passing rates**

		**2%/2 mm**		**1%/2 mm**		**2%/1 mm**	
	***d***	***r***_***s***_	***p***	***r***_***s***_	***p***	***r***_***s***_	***p***
ASM	1	0.522	0.001	0.603	<0.001	0.469	0.002
	5	0.421	0.007	0.496	0.001	0.356	0.024
	10	0.389	0.013	0.489	0.001	0.334	0.035
IDM	1	−0.504	0.001	−0.605	<0.001	−0.451	0.003
	5	−0.528	<0.001	−0.571	<0.001	−0.494	0.001
	10	−0.462	0.003	−0.528	<0.001	−0.402	0.010
Contrast	1	0.588	<0.001	0.640	<0.001	0.644	<0.001
	5	0.593	<0.001	0.629	<0.001	0.613	<0.001
	10	0.603	<0.001	0.642	<0.001	0.584	<0.001
Variance	1	0.519	0.001	0.525	0.001	0.594	<0.001
	5	0.538	<0.001	0.538	<0.001	0.599	<0.001
	10	0.464	0.003	0.477	0.002	0.549	<0.001
Correlation	1	−0.473	0.002	−0.585	<0.001	−0.458	0.003
	5	−0.565	<0.001	−0.633	<0.001	−0.492	0.001
	10	−0.561	<0.001	−0.605	<0.001	−0.430	0.006
Entropy	1	−0.467	0.002	−0.527	<0.001	−0.470	0.002
	5	−0.447	0.004	−0.529	<0.001	−0.437	0.005
	10	−0.443	0.004	−0.534	<0.001	−0.418	0.007

*Abbreviations:* d = particular displacement distance, r_s_ = Spearman’s rank correlation coefficient, ASM = angular second moment, IDM = inverse difference moment.

### Correlations between textural features and MLC positional errors

The *r*_*s*_ values and corresponding *p* values of textural features to the differences in mechanical parameters are shown in Table 4. No statistically significant correlations were observed between textural features and the differences in MU.

**Table 4:** **The values of r**_**s**_ **between textural features and mechanical parameter differences**

		**MLC error**		**Gantry angle error**		**MU error**	
	***d***	***r***_***s***_	***p***	***r***_***s***_	***p***	***r***_***s***_	***p***
ASM	1	−0.747	<0.001	0.445	0.004	0.108	0.506
	5	−0.749	<0.001	0.499	0.001	0.165	0.309
	10	−0.785	<0.001	0.600	<0.001	0.106	0.513
IDM	1	0.604	<0.001	−0.444	0.004	−0.243	0.131
	5	0.821	<0.001	−0.705	<0.001	−0.217	0.179
	10	0.787	< 0.001	−0.716	< 0.001	−0.206	0.203
Contrast	1	−0.853	< 0.001	0.655	< 0.001	0.145	0.372
	5	−0.843	< 0.001	0.643	< 0.001	0.160	0.323
	10	−0.825	< 0.001	0.636	< 0.001	0.217	0.179
Variance	1	−0.699	< 0.001	0.526	< 0.001	0.110	0.501
	5	−0.690	< 0.001	0.510	0.001	0.090	0.579
	10	−0.648	< 0.001	0.507	0.001	0.040	0.808
Correlation	1	0.766	< 0.001	−0.510	0.001	−0.050	0.761
	5	0.758	< 0.001	−0.540	< 0.001	−0.132	0.416
	10	0.728	< 0.001	−0.533	< 0.001	−0.157	0.332
Entropy	1	0.820	< 0.001	−0.516	0.001	−0.095	0.559
	5	0.823	< 0.001	−0.538	< 0.001	−0.169	0.297
	10	0.841	< 0.001	−0.592	< 0.001	−0.117	0.474

*Abbreviations:* MLC = multi-leaf collimator, MU = monitor unit, d = particular displacement distance, r_s_ = Spearman’s rank correlation coefficient, ASM = angular second moment, IDM = inverse difference moment.

For MLC positional errors, the highest *r*_*s*_ value was observed between *contrast* (*d* = 1) and MLC errors (*r*_*s*_ = -0.853 with *p* < 0.001). In the case of *contrast* (*d* = 1) with edge-enhanced fluences, the *r*_*s*_ value to MLC errors was smaller than that of *contrast* (*d* = 1) with non-edge-enhanced fluences (*r*_*s*_ = -0.863 with *p* < 0.001), LTMCS (*r*_*s*_ = -0.857 with *p* < 0.001) and MI_t_ (*r*_*s*_ = 0.917 with *p* < 0.001) while it was larger than that of *variance* (*d* = 1) with non-edge-enhanced fluences (*r*_*s*_ = -0.828 with *p* < 0.001), MCS_v_ (*r*_*s*_ = -0.635 with *p* < 0.001) and MI_SPORT_ (*r*_*s*_ = 0.795 with *p* < 0.001).

For gantry angle errors, the highest correlation was observed between *IDM* (*d* = 10) and gantry angles (*r*_*s*_ = -0.716 with *p* < 0.001). In the case of *contrast* (*d* = 1) with edge-enhanced fluences, *r*_*s*_ value to gantry angle error (0.655 with *p* < 0.001) was smaller than those of LTMCS (*r*_*s*_ = -0.714 with *p* < 0.001) and MI_SPORT_ (*r*_*s*_ = 0.721 with *p* < 0.001) while it was larger than those of MCS_v_ (*r*_*s*_ = -0.620 with *p* < 0.001), MI_t_ (*r*_*s*_ = 0.630 with *p* < 0.001) and *contrast* (*d* = 1) and *variance* (*d* = 1) with non-edge-enhanced fluences (*r*_*s*_ = 0.639 with *p* < 0.001 and *r*_*s*_ = 0.628 with *p* < 0.001, respectively) [23].

### Correlations between textural features and dose-volumetric parameters

The statistically significant *r*_*s*_ values of textural features calculated with edge-enhanced fluences generated from prostate and H&N VMAT plans to differences in the clinically relevant dose-volumetric parameters between plan and delivery are shown in Tables 5 and 6, respectively. Statistically significant values of *r*_*s*_ were found more frequently between *variance* (*d* = 1, 5 and 10) and the differences in dose-volumetric parameters (13 cases from a total of 35 cases), than between other textural features and the dose-volumetric differences. *Contrast* (*d* = 1) showed statistically significant *r*_*s*_ values in 11 cases to the dose-volumetric parameter differences. The numbers of statistically significant *r*_*s*_ values to the dose-volumetric parameter differences of MCS_v_, LTMCS, MI_SPORT_, MI_t_ and *contrast* (*d* = 1) and *variance* (*d* = 1) with non-edge-enhanced fluences were 3, 2, 4, 15, 4 and 10, respectively [23]. Therefore, the performance of *contrast* (d = 1) with edge-enhanced fluences was better than those of MCS_v_, LTMCS, MI_SPORT_, *contrast* (*d* = 1) and *variance* (*d* = 1) with non-edge-enhanced fluences while it was inferior to that of MI_t_.

**Table 5:** **The values of statistically significant r**_**s**_ **of textural features to dose-volumetric parameter differences of prostate VMAT plans**

	**Contrast**				**Correlation**			
	***d*** **= 1**		***d*** **= 10**		***d*** **= 1**		***d*** **= 10**	
	***d*** **= 1**		***d*** **= 10**		***d*** **= 1**		***d*** **= 10**	
Dose-volumetric parameter	*r*_*s*_	*p*	*r*_*s*_	*p*	*r*_*s*_	*p*	*r*_*s*_	*p*
D20% of rectal wall	-	-	-	-	0.485	0.030	-	-
Mean dose to rectal wall	−0.493	0.027	−0.473	0.035	0.446	0.048	-	-
Mean dose to bladder	−0.456	0.043	-	-	-	-	-	-
D50% of femoral heads	-	-	-	-	-	-	0.448	0.047

*Abbreviations:* d = particular displacement distance, r_s_ = Spearman’s rank correlation coefficient, Dn% = dose received by n% volume of structure.

**Table 6:** **The values of statistically significant r**_**s**_ **of textural features to dose-volumetric parameter differences of head and neck VMAT plans**

	***d*** **= 1**		***d*** **= 5**		***d*** **= 10**	
	***r***_***s***_	***p***	***r***_***s***_	***p***	***r***_***s***_	***p***
	ASM					
D_5%_ of target 2	-	-	0.501	0.026	-	-
Mean dose to target 2	0.532	0.016	0.565	0.009	-	-
D_95%_ of target 3	-	-	0.641	0.003	-	-
D_5%_ of target 3	0.558	0.013	0.635	0.003	-	-
Mean dose to target 3	0.489	0.034	0.635	0.003	-	-
	IDM					
D_5%_ of target 1	-	-	0.511	0.021	0.478	0.033
Mean dose to target 1	-	-	0.562	0.010	0.520	0.019
D_95%_ of target 2	-	-	0.490	0.028	-	-
Minimum dose to target 2	0.447	0.048	-	-	-	-
Mean dose to target 2	0.501	0.024	0.593	0.006	0.548	0.012
D_5%_ of target 3	-	-	0.512	0.025	-	-
Mean dose to right parotid gland	0.540	0.014	0.619	0.004	-	-
Mean dose to left parotid gland	-	-	0.506	0.023	-	-
Maximum dose to right optic nerve	0.498	0.025	0.517	0.020	-	-
	Contrast					
D_5%_ of target 1	-	-	−0.454	0.044	−0.502	0.024
Mean dose to target 1	−0.486	0.030	−0.539	0.014	−0.488	0.029
D_95%_ of target 2	−0.494	0.027	−0.651	0.002	-	-
D_5%_ of target 2	−0.534	0.017	−0.618	0.004	−0.477	0.035
Minimum dose to target 2	−0.478	0.033	-	-	-	-
Mean dose to target 2	−0.626	0.003	−0.704	0.001	−0.521	0.018
D_95%_ of target 3	-	-	−0.508	0.026	-	-
D_5%_ of target 3	−0.576	0.010	−0.635	0.003	-	-
Mean dose to target 3	−0.484	0.036	−0.493	0.032	-	-
Mean dose to right parotid gland	−0.595	0.006	−0.516	0.020	-	-
Maximum dose to right optic nerve	−0.631	0.003	−0.477	0.033	-	-
	Variance					
D_95%_ of target 1	−0.553	0.012	−0.558	0.011	−0.589	0.006
D_5%_ of target 1	−0.693	0.001	−0.689	0.001	−0.688	0.001
Mean dose to target 1	−0.705	0.001	−0.708	< 0.001	−0.727	< 0.001
D_95%_ of target 2	−0.630	0.003	−0.617	0.004	−0.626	0.003
D_5%_ of target 2	−0.668	0.002	−0.672	0.002	−0.645	0.003
Mean dose to target 2	−0.734	< 0.001	−0.733	< 0.001	−0.734	< 0.001
D_95%_ of target 3	−0.553	0.014	−0.548	0.015	−0.519	0.023
D_5%_ of target 3	−0.665	0.002	−0.670	0.002	−0.691	0.001
Maximum dose to target 3	−0.511	0.025	−0.490	0.033	−0.497	0.030
Mean dose to target 3	−0.487	0.035	−0.502	0.029	−0.519	0.023
Mean dose to right parotid gland	−0.705	0.001	−0.675	0.001	−0.709	< 0.001
Mean dose to left parotid gland	−0.479	0.033	−0.444	0.050	−0.458	0.042
Maximum dose to right optic nerve	−0.568	0.009	−0.560	0.010	−0.560	0.010
	Correlation					
D_5%_ of target 1	−0.589	0.006	−0.546	0.013	-	-
Maximum dose to target 1	-	-	−0.490	0.028	-	-
	Entropy					
D_5%_ of target 1	-	-	−0.487	0.029	-	-
Mean dose to target 1	-	-	−0.466	0.038	-	-
D_5%_ of target 2	-	-	−0.519	0.021	−0.451	0.047
Mean dose to target 2	−0.465	0.039	−0.537	0.015	-	-
D_95%_ of target 3	−0.522	0.022	−0.584	0.009	−0.497	0.030
D_5%_ of target 3	−0.598	0.007	−0.656	0.002	−0.543	0.016
Mean dose to target 3	−0.487	0.035	−0.552	0.014	-	-

*Abbreviations:* d = particular displacement distance, r_s_ = Spearman’s rank correlation coefficient, Dn% = dose received by n% volume of structure, ASM = angular second moment, IDM = inverse difference moment.

## Discussion

In a previous study, we demonstrated the potential of textural features calculated from fluences generated from VMAT plans as a modulation index, showing considerable correlations to VMAT delivery accuracy as quantified with gamma-index method, quantification of mechanical parameter differences between plan and delivery using linac log file and analysis on the differences in dose-volumetric parameters between plan and delivery with linac log files [23]. In that study, *contrast* (*d* = 1) and *variance* (*d* = 1) showed stronger correlations to VMAT delivery accuracy as compared to MCS_v_, LTMCS and MI_SPORT_. However, as mentioned above, the effect of some small or irregular fields on the values of textural features might be smeared out because every MU map was simply integrated to generate the fluences in our previous study. Therefore, we doubled the values in the pixels representing MLC tips in a fluence to identify small or irregular fields in a single fluence, and performed correlation analysis between the textural features calculated from that fluence and VMAT delivery accuracy in this study. We found generally stronger correlations of *contrast* (*d* = 1) to VMAT delivery accuracy than those of textural features in our previous study, as well as conventional modulation indices. By enhancement of values in the region of MLC tips at each CP in a fluence, we improved the performance of *contrast* (*d* = 1) as a modulation index for VMAT.

Just as in our previous study, the values of *contrast* (*d* = 1) of lowly-modulated VMAT plans (prostate VMAT plans) were higher than those of highly-modulated VMAT plans (H&N VMAT plans) [23]. Due to the enhancement of values in the region of MLC tips in this study, values of *contrast* (*d* = 1) of both prostate and H&N VMAT plans increased compared to those calculated with non-enhanced fluences. The most noticeable improvement of *contrast* (*d* = 1) by edge-enhancement of fluences were observed in the number of statistically significant *r*_*s*_ values to the differences in dose-volumetric parameters (4 cases with non-edge-enhancement vs. 11 cases with edge-enhancement) [23]. Besides that, performance improvements of *contrast* (*d* = 1) by edge-enhancement were observed in both global and local gamma passing rates with every gamma criterion tested in this study and gantry angle errors. Although a lower value of *r*_*s*_ was observed between *contrast* (*d* = 1) and MLC errors by edge-enhancement of fluences (-0.853 with edge-enhanced fluences vs. -0.863 with non-edge-enhanced fluences), *contrast* (*d* = 1) still showed strong correlation to MLC errors, with a value higher than 0.8 (*p* < 0.001). Comparing *contrast* (*d* = 1) with edge-enhancement and *variance* (*d* = 1) with non-edge-enhancement, with the exception of correlation to global gamma passing rates with 2%/2 mm, *contrast* (*d* = 1) with enhancement always showed stronger correlations than did *variance* (*d* = 1) with non-enhancement to every method of VMAT delivery accuracy verification tested in this study [23]. *Contrast* (*d* = 1) with enhancement always showed stronger correlations to plan delivery accuracy than did MCS_v_ [23]. In the case of LTMCS, with the exception of correlations to mechanical parameter differences, *contrast* (*d* = 1) with enhancement of fluences showed stronger correlations to every method of plan delivery accuracy verification [23]. For MI_SPORT_, *contrast* (*d* = 1) showed stronger correlations to every method of plan delivery accuracy verification except correlation to gantry angle errors [23]. To compare *contrast* (*d* = 1) with edge-enhancement to MI_t_, better performance was shown by *contrast* (*d* = 1) in global gamma passing rates with 2%/2 mm and 2%/1 mm, local gamma passing rates with 2%/1 mm and gantry angle errors than MI_t_, while it showed inferior performance in global gamma passing rates with 1%/2 mm, local gamma passing rates with 2%/2 mm and 1%/2 mm, MLC errors and number of statistically significant dose-volumetric parameters between plan and delivery than MI_t_ [6]. Since we quantified plan delivery accuracy with various verification methods and those results were not always consistent in this study, similar to the findings of Nelms *et al.* (data are not shown), neither *contrast* (*d* = 1) nor MI_t_ always showed stronger correlations to the results of every method of plan delivery accuracy verification [16]. To determine which indicator is superior, further analysis by increasing sample size and collecting various types of samples (VMAT plans generated with various types of TPS, linacs or treatment sites and gamma evaluation with various types of detectors) should be done. This will be performed as a future work.

We acquired gamma passing rates using a single detector array (MapCHECK2™ detector array) which has a spatial resolution of 7.07 mm. The insufficient spatial resolution might cause weak correlations between gamma passing rates and the textural features in this study. However, we believe that those weak correlations came from the intrinsic limitation of the 2D gamma-index method rather than the poor resolution of MapCHECK2™ detector array considering the study by Kim *et al* [15]. As mentioned above, further study on this adopting various types of detectors will be done as a future work.

We could not suggest a tolerance level for *contrast* (*d* = 1) with edge-enhanced fluences in this study since the sample size was only 40. Moreover, all the VMAT plans in this study were clinically acceptable. Since no excessively-modulated VMAT plans which were clinically unacceptable were included in this study, we couldn’t acquire tolerance level for *contrast* (*d* = 1) to identify clinically unacceptable VMAT plans. As mentioned above, by increasing samples with various types of VMAT plans and by the inclusion of excessively-modulated VMAT plans, a tolerance level for *contrast* (*d* = 1) with edge-enhanced fluences will be suggested as a future work.

As shown in our previous study, the variations of gamma passing rates, mechanical parameter differences between plan and delivery and dose-volumetric parameter differences were small, as every VMAT plan in this study was clinically acceptable and used for patient treatment [23]. The global gamma passing rates with 2%/2 mm criterion recommended by Heilemann *et al.* for VMAT pre-treatment QA were 98.6% for prostate VMAT plans and 97.0% for H&N VMAT plans on average [13]. The mean errors in MLC positions, gantry angles and MUs were 0.24 mm, 0.39° and 0.16 MU, respectively, for prostate VMAT plans and 0.80 mm, 0.37° and 0.14 MU, respectively, for H&N VMAT plans, showing minimal differences. Within this small variation, *contrast* (*d* = 1) with edge-enhanced fluences showed considerable correlations with statistical significances to every type of verification method for VMAT delivery accuracy. Therefore, *contrast* (*d* = 1) with edge-enhanced fluences could be used as a modulation index for VMAT and it could possibly reject highly-modulated VMAT plans at the planning stage.

We could not guarantee accuracy of patient treatment with only the value of *contrast* (*d* = 1) since there are various factors affecting patient treatment accuracy, such as patient respiratory motion, setup uncertainty and anatomy changes during treatment. Although we could not predict treatment accuracy by evaluating the value of *contrast* (*d* = 1), at least, we could predict the delivery accuracy of VMAT using that value. We believe this has some value in the clinic.

## Conclusions

*Contrast* (*d* = 1) calculated from fluences with enhancement of values at the tips of MLCs to prevent potential smearing out of small or irregular fields showed considerable correlations with statistical significances to gamma passing rates, mechanical errors during delivery and differences in dose-volumetric parameters between plan and delivery of VMAT. It showed stronger correlations to plan delivery accuracy than previously suggested textural features, including *contrast* (*d* = 1) and *variance* (*d* = 1) with non-edge-enhancement as well as MCS_v_, LTMCS and MI_SPORT_ [23]. *Contrast* (*d* = 1) with edge-enhancement could be used as a modulation index for VMAT to predict plan delivery accuracy.

## Abbreviations

VMAT: Volumetric modulated arc therapy
MLC: Multi-leaf collimator
IMRT: Intensity-modulated radiation therapy
TPS: Treatment planning system
QA: Quality assurance
MCS_v_: Modulation complexity score for volumetric modulated arc therapy
LTMCS: Leaf travel modulation complexity score
MCS: Modulation complexity score
MI_SPORT_: Modulation index supporting station parameter optimized radiation therapy
MU: Monitor unit
CP: Control point
MI_t_: Modulation index of total consideration of modulating parameters of volumetric modulated arc therapy
H&N: Head and neck
PRO3: Progressive resolution optimizer 3
AAA: Anisotropic analytic algorithm
SIB: Simultaneously integrated boost
GLCM: Gray level co-occurrence matrix
d: Particular displacement distance
ASM: Angular second moment
IDM: Inverse difference moment
OAR: organ at risk
AAPM: American association of physicists in medicine
TG: Task group
CBCT: Cone beam computed tomography
D_n%_: Dose received by n% volume of a structure
r_s_: Spearman’s rank correlation coefficient
